# Using predictions from a joint model for longitudinal and survival data to inform the optimal time of intervention in an abdominal aortic aneurysm screening programme

**DOI:** 10.1002/bimj.201600222

**Published:** 2017-04-24

**Authors:** Michael J. Sweeting

**Affiliations:** ^1^ Department of Public Health and Primary Care Cardiovascular Epidemiology Unit Worts Causeway Cambridge CB1 8RN UK

**Keywords:** Abdominal aortic aneurysm, Decision making, Dynamic predictions, Joint modelling, Personalised medicine

## Abstract

Joint models of longitudinal and survival data can be used to predict the risk of a future event occurring based on the evolution of an endogenous biomarker measured repeatedly over time. This has led naturally to the use of dynamic predictions that update each time a new longitudinal measurement is provided. In this paper, we show how such predictions can be utilised within a fuller decision modelling framework, in particular to allow planning of future interventions for patients under a ‘watchful waiting’ care pathway. Through the objective of maximising expected life‐years, the predicted risks associated with not intervening (e.g. the occurrence of severe sequelae) are balanced against risks associated with the intervention (e.g. operative risks). Our example involves patients under surveillance in an abdominal aortic aneurysm screening programme where a joint longitudinal and survival model is used to associate longitudinal measurements of aortic diameter with the risk of aneurysm rupture. We illustrate how the decision to intervene, which is currently based on a diameter measurement greater than a certain threshold, could be made more personalised and dynamic through the application of a decision modelling approach.

## 1 Introduction

Joint models of longitudinal and survival data are now used widely to understand the relationship between a time‐varying endogenous process and the occurrence of an event of interest, e.g. CD4 cell count and the onset of AIDS (Tsiatis et al., 1995); PSA antigen and prostate cancer (Yu et al., 2008); and abdominal aortic aneurysm (AAA) size and risk of rupture (Sweeting and Thompson, 2011). Another important use of such models is for prediction, and in particular individualised dynamic predictions, whereby predictions are updated as more longitudinal data accrues on the individual, of which there are a number of examples in the literature (Yu et al., 2008; Proust‐Lima and Taylor, 2009; Rizopoulos, 2011; Andrinopoulou et al., 2015).

In this paper, we show how such predictions obtained from a joint model can be utilised together with external data sources to inform the time at which an intervention should be made due to the risk of the event of interest becoming too great. Such a question is relevant to diseases such as AAA or prostate cancer, where one available pathway of care for patients determined to be at low risk is a ‘waitful watching’ approach. Here patients undergo regular monitoring, and an intervention such as surgery or radiation treatment is planned only if the disease is deemed to have progressed sufficiently.

Our motivating example involves patients with a small AAA who are regularly monitored within a screening programme (such as the UK National Aortic Aneurysm Screening Programme) using ultrasound scans. AAAs are swellings of the aorta that can grow over time and if left untreated can rupture, with an associated high rate of mortality. Within a screening programme, at each visit, a patient's AAA diameter is measured and recorded. Diameters between 3.0 and 5.4 cm are considered small with the risk of the aneurysm rupturing being relatively low, and hence the patient is often advocated to continue surveillance. Evidence for this approach comes from two large randomised trials (Lederle et al., 2002; United Kingdom Small Aneurysm Trial Participants, 2002), which showed no advantage for early surgery and continued surveillance being more cost‐effective. Once the AAA is measured greater than or equal to 5.5 cm, patients are generally considered for elective surgery (either open abdominal or endovascular surgery), since it is then presumed that the risk of rupture outweighs any operative risk. However, the evidence to suggest operating at 5.5 cm rather than waiting longer is rather weak. Furthermore, for some patients it may be better to intervene earlier than others due to a more favourable operative risk or a less favourable rupture risk.

We will illustrate, through utilisation of a joint model for AAA growth and rupture risk, how to estimate an optimal time of surgery based on a trade‐off between the predicted rupture risk and operative risk. Optimality will be defined based on maximising expected life‐years. We will show that such an approach can allow predictions to be updated dynamically over time as more information on AAA size and rate of growth accrues, thus allowing forward planning regarding the likely timing of an operation. Furthermore, we show that the modelling approach can be easily extended to make predictions even more personalised, by, for example, taking the age of the patient into account, to acknowledge that operative risks generally increase with age. From the application of the joint model to data from the Multicentre Aneurysm Screening Study (MASS; Ashton et al., 2002) and utilisation of external data on operative risk, we make some initial recommendations regarding the optimal intervention time and diameter threshold for elective surgery.

## 2 A joint longitudinal and survival model for AAA growth and rupture

### 2.1 The model

We follow a joint modelling approach to relate the evolution of an aneurysm (e.g. its diameter and rate of growth) to the risk of rupture, as has previously been proposed (Sweeting and Thompson, 2011; Thompson et al., 2013). Let $T_{i}$ be the observed event time for the *i*‐th patient, i=1,…,n, where $T_{i}=\min\left(T_{i}^{*},C_{i}\right)$ is the minimum of the true event time and a censoring time $C_{i}$, and $\delta_{i}=I\left(T_{i}^{*}\leq C_{i}\right)$ the event indicator. In this application, the event time is an occurrence of a fatal or non‐fatal ruptured AAA. Let $y_{i}=\left\{y_{i}\left(t_{ij}\right),j=1,\ldots ,n_{i}\right\}$ denote a set of AAA diameters, measured via ultrasound scans, for patient *i* at observation times $\left\{t_{ij},j=1,\ldots ,n_{i}\right\}$ since entering the study. As the diameter is measured with error, we relate the observed AAA diameters to an underlying process (a patient‐specific trajectory) using a linear mixed‐effects model:

$$\begin{array}{ccc} y_{i}\left(t_{ij}\right) & = & m_{i}\left(t_{ij}\right)+\varepsilon_{ij},\;\;\;\varepsilon_{ij}∼N\left(0,\sigma_{\varepsilon }^{2}\right)\\ m_{i}\left(t_{ij}\right) & = & x_{i}^{T}\left(t_{ij}\right)\beta +z_{i}^{T}\left(t_{ij}\right)b_{i}, \end{array}$$

where $m_{i}\left(t\right)$ is the true and unobserved longitudinal trajectory for patient *i* and **x** and **z** are design matrices for the fixed effects (β) and random effects ($b_{i}$), respectively. The random effects $b_{i}$ are assumed normally distributed with zero mean and covariance matrix Σ and are independent of the errors $\varepsilon_{ij}$. The exact form of the trajectory function will be determined by the observed data; previously, we have found that a linear model is an adequate approximation of AAA growth over a short time period (e.g. a couple of years; Sweeting and Thompson, 2012), although the rate of growth does increase steadily over time, and hence a quadratic or more complex functional form may be more realistic.

For the survival sub‐model, the risk of a rupture is related to a set of baseline covariates **w** and the AAA trajectory function $m_{i}\left(t\right)$ as follows:

$$\begin{array}{ccc} h_{R,i}\left(t\right) & = & h_{R,0}\left(t\right)\;\exp\left(\gamma^{T}w_{i}+\alpha f\left(m_{i}\left(t\right)\right)\right), \end{array}$$

where $h_{R,0}\left(\cdot \right)$ is a baseline hazard function and α is a vector of association parameters, which relate the longitudinal outcome process and/or the random effects to the time‐to‐event outcome of interest (rupture). A number of choices for the functional form of f(·) have been proposed (Rizopoulos, 2012). In this paper, we will consider just three, based on the previous investigations of the relationship between AAA diameter and rupture risk (Sweeting and Thompson, 2011), namely

1. $\alpha f\left(m_{i}\left(t\right)\right)=\alpha m_{i}\left(t\right)$ : an association with the current AAA diameter;
2. $\alpha f\left(m_{i}\left(t\right)\right)=\alpha_{1}m_{i}\left(t\right)+\alpha_{2}m_{i}^{\prime }\left(t\right)$, where $m_{i}^{\prime }\left(t\right)=\frac{d}{dt}m_{i}\left(t\right)$ : an association with both current AAA diameter and rate of change;
3. $\alpha f\left(m_{i}\left(t\right)\right)=\alpha_{1}m_{i}\left(t\right)+\alpha_{2}\int_{0}^{t}m_{i}\left(s\right)ds$ : an association with both current AAA diameter and area under the AAA trajectory from the time of entering the study.

Define $\theta =\left\{\theta_{t},\theta_{y},\theta_{b}\right\}$ to be the full parameter vector with subcomponents relating to the survival process, $\theta_{t}$; the longitudinal process, $\theta_{y}$ and the random‐effects, $\theta_{b}$. The likelihood is formed from the assumption of full conditional independence between the longitudinal and survival sub‐models conditional on the random effects and the contribution from patient *i* can be written as

$$\begin{array}{ccc} L_{i} & = & \int p\left(y_{i}|b_{i},\theta_{y}\right)p\left(T_{i},\delta_{i}|b_{i},\theta_{t}\right)p\left(b_{i}|\theta_{b}\right)\;db_{i}=\\ & = & \left.\int \left[\prod_{j=1}^{n_{i}}p\left(y_{ij}|b_{i},\theta_{y}\right)\right]h_{R,i}{\left(T_{i}|b_{i},\theta_{t}\right)}^{\delta_{i}}S_{R,i}\left(T_{i}|b_{i},\theta_{t}\right)p\left(b_{i}|\theta_{b}\right)\;db_{i}\right., \end{array}$$

where $S_{R,i}\left(t\right)=\exp\left(-\int_{0}^{t}h_{R,i}\left(s\right)ds\right)$ is the survivor‐like function for a rupture event. The likelihood can be maximised using adaptive Gauss–Hermite quadrature (Rizopoulos, 2012; Crowther et al., 2016) as, for example, implemented in the R package JM.

### 2.2 Predicted risk of rupture

The joint model can be used to make a prediction for an individual *i* of the probability of rupture by a future time *u* given no rupture has occurred by time *t* (ignoring any competing risks). The individual has a set of previous AAA diameter measurements $Y_{i}\left(t\right)=\left\{y_{i}\left(s\right);0\leq s\leq t\right\}$ and baseline covariates $w_{i}$. This conditional probability is given by

(1) $$\begin{array}{ccc} & & F_{R,i}(u\left|t,Y_{i}\left(t\right),w_{i},\theta )=P(T_{i}^{*}\leq u\right|T_{i}^{*}>t,Y_{i}\left(t\right),w_{i};\theta )=\\ & = & 1-\int P(T_{i}^{*}>u\left|T_{i}^{*}>t,b_{i},w_{i};\theta_{t})p(b_{i}\right|T_{i}^{*}>t,Y_{i}\left(t\right),w_{i};\theta )\;db_{i}=\\ & = & 1-\int \frac{S_{R,i}\left(u\right|b_{i},w_{i};\theta_{t})}{S_{R,i}\left(t\right|b_{i},w_{i};\theta_{t})}p(b_{i}|T_{i}^{*}>t,Y_{i}\left(t\right),w_{i};\theta )\;db_{i}. \end{array}$$

To evaluate (1), Rizopoulos (2011) proposed an asymptotic Bayesian formulation using a Monte‐Carlo simulation scheme in order to derive the posterior expectation of (1) and to compute confidence intervals (CIs) using the sample percentiles.

## 3 Calculating optimal expected life‐years

Given a predicted survival function for time to rupture, the question that arises is how to utilise this information to decide when to surgically intervene. To do this, a decision rule is set up based solely on maximising expected life‐years for illustration of the key ideas. However, the methodology could easily be extended to consider additional aspects such as costs and pre‐ and post‐operative quality of life. The methods proposed here could also be incorporated into a larger economic decision model, such as a discrete event simulation model (Brennan et al., 2006; Meester et al., 2015).

Expected life‐years can be calculated as the area under the overall survival curve. Before an intervention takes place, the survivor function relates to both AAA‐related causes of death (i.e. from rupture) and non‐AAA‐related causes of death. After elective surgical intervention (using endovascular or open repair of the intact AAA), the survivor function is assumed to be composed of deaths from immediate (or near‐immediate) peri‐operative AAA‐related causes, and longer term non‐AAA‐related causes of death (although longer term AAA‐related causes occurring due to complications with the stent graft could also be included in the modelling). Given an elective intervention at time $T_{x}$, define $p_{E,i}\left(w_{i},T_{x}\right)$ to be the peri‐operative risk of dying from an elective operation (usually defined to be the 30‐day or in‐hospital mortality probability) at time $T_{x}$ and $p_{R,i}\left(w_{i}\right)$ to be the short‐term (e.g. 30‐day) probability of mortality given a rupture occurs, both of which could depend on baseline covariates $w_{i}$. The probability of death following rupture is composed of both mortality before an emergency operation can take place and the peri‐operative mortality risk given emergency surgery is undertaken. Suppose patient *i* has survived without an elective operation up to time *t*, with set of previous AAA diameter measurements $Y_{i}\left(t\right)$ and baseline covariates $w_{i}$. The expected remaining life‐years given an elective intervention at time $T_{x}>t$ can be approximated by

(2) E[ Life − years i|Tx>t,Yi(t),wi,θ]==∫tTxSi(u|t,Yi(t),wi,θ)du︸Beforeintervention+(1−pE,i(wi))∫Tx∞S nonAAA ,i(v|t,wi)dv︸Afterintervention,

where Si(u|t,Yi(t),wi,θ)=S nonAAA ,i(u|t,wi)S AAA ,i(u|t,Yi(t),wi,θ) is the overall survivor function pre‐operation composed of the non‐AAA and AAA survivor‐like functions, S nonAAA ,i(u|t,wi) and S AAA ,i(u|t,Yi(t),wi,θ), respectively. The AAA‐related survivor‐like function can be calculated from the joint model predicted risk of rupture, Eq. (1), and $p_{R,i}\left(w_{i}\right)$:

S AAA ,i(u|t,Yi(t),wi,θ)=1−FR,i(u|t,Yi(t),wi,θ)pR,i(wi).

The non‐AAA‐related survivor‐like function can be estimated from a standard survival analysis model since it is not assumed to be related to the endogenous longitudinal marker. Alternatively, estimates could be obtained from external data sources (e.g. population statistics).

The optimal intervention time $T_{x,opt}$ is then defined as the intervention time that maximises Eq. (2). In the next section, we illustrate how this can be evaluated using a finite grid of possible future intervention times.

## 4 Application to data from the MASS

Data on the growth and rupture rates of AAA come from MASS (Ashton et al., 2002), which enrolled men aged 65–74 to randomly receive an invitation for an abdominal ultrasound scan or not. Of the 33,839 men invited to screening, 1333 AAAs were detected of which 1167 had a small AAA (3.0–5.4 cm). The AAA diameters of these men were monitored over time using ultrasound, either annually (3.0–4.4 cm) or 3‐monthly (4.5–5.4 cm), until one of the following events occurred: (i) the AAA diameter was observed to be ⩾5.5 cm, (ii) the patient's AAA ruptured, (iii) the patient died from non‐AAA‐related causes, (iv) the patient was lost to follow‐up or (v) the patient reached the end of the study follow‐up period. Patients who were observed to have a ⩾5.5 cm AAA at any point during follow‐up were considered for elective surgery and hence for the majority of individuals, the first ultrasound scan ⩾5.5 cm was their last recorded diameter. These patients have a series of AAA diameter measurements that are censored due to crossing a pre‐defined threshold; such a mechanism is not problematic since it causes data to be missing at random. For a minority of individuals (21%), further AAA diameter measurements were recorded after their first measurement ⩾5.5 cm—some of these individuals were considered unfit (or contraindicated) for surgery. These additional diameter measurements are not used in the primary analysis of AAA growth, since these patients may not provide representative estimates of growth beyond 5.5 cm. However, this does mean that determining rupture risk for AAAs >5.5 cm must be based entirely on model extrapolation. As a sensitivity analysis, we therefore consider including the extended follow‐up data on those deemed unfit for surgery (see Section 4.5).

The analysis is restricted to data on 1122 individuals whose initial AAA diameter measured in the anterior–posterior plane (inner to inner luminal surface) was between 3.0 and 5.4 cm (Thompson et al., 2013). Patients were followed up for a mean of 5.4 years until either rupture or censoring (due to one of the reasons listed above); 33 ruptures occurred during 6085 person‐years of observation. A second dataset, based on the data from the UK Small Aneurysm Trial and Study (UKSAT; United Kingdom Small Aneurysm Trial Participants, 2002; Brady et al., 2004), is used to externally validate the fitted models. These data contain 2227 patients with an aneurysm followed up for an average of 2.4 years, in which 60 ruptures occurred. In this study, AAA diameters were measured via ultrasound using an outer to outer aortic wall measurement, which on average gives a larger reading of the diameter, and hence the data are less relevant for making recommendations regarding thresholds for the UK National Aortic Aneurysm Screening Programme, which is based on inner to inner measurements. Nevertheless, it is still of interest to see how well our fitted model performs on these data.

### 4.1 Results from the joint AAA growth and rupture model

The joint AAA growth and rupture model is fit to the MASS data using time since study entry as the underlying time scale and AAA diameter, measured in millimetres, as the longitudinal outcome. Table 1 shows the log‐likelihood and Akaike information criterion (AIC) from a number of parameterisations of the joint model. A large decrease in AIC is observed when the AAA trajectory is modelled using a quadratic function of time (with random intercept and random linear term) compared to a linear function of time. A further decrease in AIC is observed when using both current value and slope or current value and cumulative effect as association variables. There is no improved fit when using a Weibull baseline hazard over an exponential hazard function.

**Table 1:** Joint model selection metrics

Longitudinal	Survival		LL	AIC	DDIc)	
sub‐model	sub‐model				MASS	UKSAT
AAA trajectory:	Baseline hazard:	Association structure:				
Lineara)	Exponential	CV	−23,844	47,704	0.915 (0.893)	0.875 (0.888)
Quadraticb)	Exponential	CV	−23,735	47,489	0.910 (0.891)	0.872 (0.885)
Quadraticb)	Weibull	CV	−23,735	47,489	0.914 (0.894)	0.874 (0.888)
Quadraticb)	Exponential	Slope	−23,742	47,501	0.900 (0.873)	0.859 (0.861)
Quadraticb)	Exponential	CV & slope	−23,732	47,484	0.916 (0.895)	0.873 (0.887)
Quadraticb)	Exponential	CV & cum. effect	−23,732	47,484	0.921 (0.896)	0.882 (0.896)

*Note*. LL, log‐likelihood; AIC, Akaike information criterion; DDI, dynamic discrimination index; MASS, Multicentre Aneurysm Screening Study; UKSAT, United Kingdom Small Aneurysm Trial; CV, current value.

a) Random intercepts and slopes.

b) Random intercepts, random linear effects and fixed curvature effects.

c) Calculated over a prediction time frame of 2 years (and 5 years in parentheses).

To investigate the predictive ability of the model to discriminate between individuals who will rupture or not within a future period of time, we calculate the dynamic discrimination index (DDI) proposed by Rizopoulos (2011) as an omnibus summary measure of the area under the receiver operating characteristic (ROC) curve based on a weighted average over follow‐up . The function dynCJM in the JM package in R was used to calculate the DDI, considering a future prediction interval of either 2 years or 5 years. The predictive ability of all the models is high, with the DDI above 0.9 for 2‐year predictions in the MASS data and above 0.85 in the UKSAT. The DDI for a 5‐year prediction is slightly lower using the MASS data, but slightly higher in the UKSAT data. In contrast to the AIC measure, the DDI does not improve when the AAA trajectory is modelled using a quadratic function of time, suggesting that for predicting rupture events, a linear trajectory may be adequate. It is clear, however, that the current value of the AAA diameter is a strong predictor, with the DDI decreasing sharply in the model in which it is not included. The highest DDI is achieved when both current value and a cumulative effect are included as predictors in the survival sub‐model.

These investigations of the AIC and DDI led us to focus on a model with a quadratic trajectory, and with a survival sub‐model with exponential baseline hazard and an association with current value and slope (despite this model having a smaller DDI than the model with an association with a cumulative effect, it was felt that a current value and slope model was more interpretable). The final parameter estimates from this model are shown in Table 2. The estimated hazard ratios associated with a 1mm increase in current AAA diameter and for a 1mm/year increase in slope are 1.09 (95% CI: 1.04, 1.13) and 1.78 (95% CI: 1.22, 2.61), respectively.

**Table 2:** Parameter estimates from final joint model of AAA growth and rupture

Parameter	Description	Estimate (SE)	*p*‐value
Longitudinal process			
β_0_	Fixed intercept	36.5 (0.2)	–
β_1_	Fixed linear slope term	2.05 (0.08)	<0.0001
β_2_	Fixed quadratic slope term (curvature)	0.097 (0.006)	<0.0001
Survival process			
$\log(h_{R,0})$	Log baseline hazard	−11.0 (0.9)	–
α_1_	Log‐hazard ratio with current diameter	0.084 (0.021)	0.0001
α_2_	Log‐hazard ratio with slope	0.579 (0.193)	0.0028
Variance components			
σ_0_	Random intercept between‐subject SD	6.63	–
σ_1_	Random linear slope term between‐subject SD	1.79	–
ρ	Correlation between random intercept and linear slope term	0.567	–
σ	Residual error SD	2.91	–

### 4.2 Decision model: Data sources

We consider two decision models: Model (1), where only the non‐AAA survivor function is dependent on age, and Model (2), where the elective operative risk and the non‐AAA survivor function are both dependent on age. Table 3 presents the sources of data used to calculated expected life‐years. Data from the National Vascular Register (NVR) 2010–2012 are used to inform the elective operative mortality (Waton et al., 2013), whilst results from a ruptured AAA randomised controlled trial (IMPROVE Trial Investigators, 2014) are used to inform emergency operative mortality. Approximately 45% of ruptured AAAs are estimated to reach surgery (Lindholt et al., 2012). Data from the Office of National Statistics inform non‐AAA causes of death.

**Table 3:** Data sources and parameter estimates used in the decision models

Parameter	Source	Model 1	Model 2
AAA growth	Ashton et al. (2002)	(See Table 2)	(See Table 2)
AAA rupture	Ashton et al. (2002)	(See Table 2)	(See Table 2)
Elective operative mortality	Waton et al. (2013)	1.9%	<66: 0.6%
(in‐hospital)			66–75: 1.6%
			76–85: 2.4%
			86+: 3.0%
Emergency operative mortality	IMPROVE Trial Investigators (2014)	37%	37%
rAAA who begin emergency surgery	Lindholt et al. (2012)	45%	45%
Non‐AAA long‐term survival	ONS Non‐AAA causes 2002‐2004	(Age‐specific)	(Age‐specific)

*Note*. MASS, Multicentre Aneurysm Screening Study; NVR, National Vascular Register; rAAA, ruptured abdominal aortic aneurysm; ONS, Office of National Statistics.

### 4.3 Decision model: Implementation

For a new individual with a given longitudinal history of AAA measurements, we use the survfitJM function in the JM package in R to obtain conditional probabilities of rupture over time. These are evaluated every 3 months from the time at which the individual was last known to be alive up to age 105 years. A Monte‐Carlo simulation scheme is implemented to evaluate these survival probabilities. For each of 1000 iterations, the estimated survival probabilities are substituted within Eq. (2) along with the estimates from the external data sources, and the trapezoidal rule is used to approximate the integrals. The expected life‐years are evaluated over a grid of possible future intervention times (in 3‐month intervals up to 105 years) and the maximum expected life‐years and associated optimal intervention time found. This process is then repeated over the Monte‐Carlo simulations to obtain distributions for the optimal intervention time, which account for the uncertainty in the rupture probabilities. The associated (underlying) AAA diameter at the optimal intervention time is obtained by substituting the optimal intervention time back into the linear mixed‐effects model. Code to implement these calculations is provided in the Supporting Information based on an example dataset of aneurysm growth and rupture.

### 4.4 Decision model: Results

**Figure 1:**
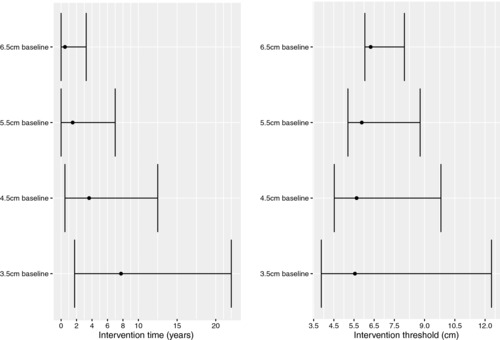
Optimal intervention times and thresholds for Model 1 given a single diameter measured at baseline (screening).

We initially consider the optimal intervention time for an individual who presents at screening with a single AAA diameter measurement. Individuals aged 65 at screening are considered with diameters of either 3.5, 4.5, 5.5 or 6.5 cm. Figure 1 presents the estimated optimal intervention time and associated diameter for these individuals for Model 1, where there is no effect of age on operative mortality. The optimal intervention time decreases rapidly as baseline diameter increases with the median recommended intervention time being less than 1 year for a 6.5 cm diameter. The precision of the estimate is relatively imprecise for a 3.5 cm diameter but increases as the diameter gets larger. The corresponding intervention threshold estimates are relatively constant across the baseline diameter measurements and vary between 5.5 and 6.3 cm.

**Figure 2:**
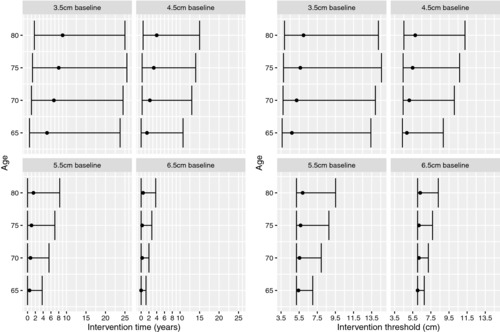
Optimal intervention times and thresholds for Model 2 given a single diameter measured at baseline (screening) and baseline age.

If age is accounted for as an operative risk factor, Model 2, then slightly different results are obtained (Fig. 2). For individuals aged 65 at screening, the optimal intervention time is almost immediate for individuals with 5.5 and 6.5 cm baseline diameters and within 1.5 years for an individual with a 4.5 cm diameter (although as previously seen the uncertainty in these estimates is high). For an 80‐year‐old, the optimal intervention threshold is relatively constant, between 6.0 and 6.3 cm depending on the observed baseline diameter. However, for a 65‐year‐old, the intervention threshold is as low as 4.7 cm for an individual with a 3.5‐cm baseline diameter, suggesting that interventions should be undertaken sooner for younger individuals. The ‘waitful watching’ approach becomes counterproductive for these younger individuals since it is more important to operate early whilst the operative risks remain relatively low.

Expected life‐year gains can be calculated and compared with the current recommended approach of operating at 5.5 cm regardless of age. These are shown in Table 4 for individuals with 3.5 cm baseline diameters. A gain in expected life‐years of 0.118 years is estimated for an individual aged 65, which translates to a percentage gain of 0.8% of their remaining life‐years. The gains in expected life‐years are small compared to those seen from deciding to intervene at 5.5 cm versus not intervening at all (Appendix Table A1). Nevertheless, further investigations are necessary to ascertain whether a more personalised intervention approach for an AAA population, which could incorporate further important clinical variables, would be both acceptable and cost‐effective.

**Table 4:** Expected life‐years remaining for individuals aged 65, 70, 75 and 80 with a 3.5 cm AAA at baseline, based on intervening at the optimal time (Model 2) and at a fixed 5.5 cm threshold

Age	Expected life‐years remaining		Difference
	Given intervention at optimum	Given intervention at 5.5 cm	
65	15.644	15.526	0.118
70	12.216	12.113	0.103
75	9.265	9.182	0.083
80	6.861	6.801	0.059

**Figure 3:**
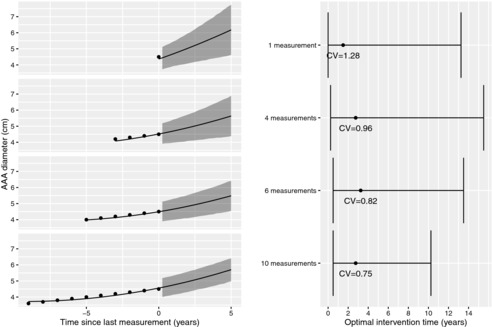
Predicted AAA trajectories and optimal intervention times for an individual aged 65 with current diameter of 4.5 cm and a series of past measurements; CV, coefficient of variation.

Dynamic predictions of the optimal intervention time can also be produced for an individual as illustrated in Fig. 3. Here, we consider an individual aged 65 with AAA size of 4.5 cm at their most recent measurement and assess how the optimal intervention time updates depending on the amount of past AAA diameter data that is taken into account. We consider using either 1, 4, 6, or 10 AAA size measurements, taken 1 year apart, all showing an observed growth rate of 1 mm/year. This is lower than the current predicted growth rate of 3.2 mm/year for the individual based on a single diameter measurement of 4.5 cm, and hence the predicted growth shrinks and the optimal intervention time lengthens as more measurements are taken into account. The 95% CIs for the optimal intervention time initially widen as more measurements are used; this is explained by the estimated optimal intervention time increasing. The coefficient of variation for the predicted optimal intervention time does decrease steadily from 1.28 using 1 measurement to 0.75 using 10 measurements. However, in this example, there is still a high degree of uncertainty even after accounting for 10 longitudinal measurements, presumably due to the uncertainty in the survival sub‐model, which was based on only 33 rupture events.

### 4.5 Sensitivity analysis: Including those contraindicated for surgery

**Figure A1:**
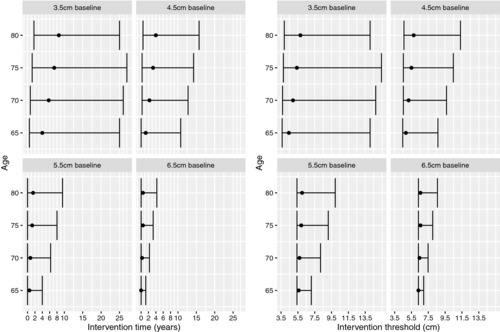
Sensitivity analysis including individuals and follow‐up time after first observed diameter ⩾5.5 cm. Optimal intervention times and thresholds Model 2 given a single diameter measured at baseline (screening) and baseline age.

Expanding the dataset to include individuals >5.4 cm at screening and those who continue surveillance after their first measurement >5.4 cm provides an extra 130 patients and an additional 28 ruptures in 455 person‐years of observation. Predictions of the optimal intervention times and thresholds under Model 2 for a single diameter measured at baseline are shown in Appendix Fig. A1 and are very similar to those presented in Fig. 2, suggesting that the results were robust to the exclusion of these additional person‐years of follow‐up.

## 5 Discussion

In this paper, we have illustrated how predictions (possibly dynamic in nature) from a joint longitudinal and survival model can be used to inform an optimal intervention time for individuals under surveillance in a AAA screening programme. We have defined optimality based on maximising expected life‐years and thus considered a direct trade‐off between short‐term elective operative mortality risk and an accumulating rupture risk. In this illustrative example, we have shown that the current UK threshold guidelines for AAA intervention (a diameter ⩾5.5 cm) are sensible for patients of older ages, but could perhaps be reduced for younger individuals. These findings are concordant with previous research, which developed a decision aid tool for management of AAA (Grant et al., 2015). However, much uncertainty remains and future work will apply these methods to data collected from multiple studies involving 11,262 men and 178 rupture events (Thompson et al., 2013).

Our model could be made more realistic by extending post‐operative AAA‐related mortality to include deaths from secondary rupture and operations undertaken to correct graft‐related complications. Such events have been shown to occur in patients undergoing endovascular surgery up to 15 years after the operation (Patel et al., 2016). It is also important to consider the health‐economic consequences of intervening at different thresholds, both in terms of changes in quality of life and the costs of the intervention and subsequent sequelae. Such consequences are currently being assessed within an individual patient decision economic model of AAA screening, which utilises a joint longitudinal and survival model similar to the one described in this paper.

Joint longitudinal and survival models are now commonplace within the statistical community, but their utility in decision modelling is only beginning to become apparent. Within the screening paradigm, joint models have been considered for obtaining personalised screening intervals (Rizopoulos et al., 2016) using information theory measures. The advantage of a joint model over a traditional Markov decision model, used routinely in health‐economic analyses, is that individual variation can be easily incorporated through the use of random‐effects and hence personalised screening strategies can be more easily assessed and implemented. The development of automated clinical decision models based on electronic health records is only likely to increase in the future and hence joint models that can dynamically update predictions based on series of longitudinal measures will inevitably become more valuable.

## Conflict of interest

*The author has declared no conflict of interest*.

## Supporting information

Supporting Information.

## Appendix 1

### 1.1

**Table A1:** Expected life‐years remaining for individuals aged 65, 70, 75 and 80 with a 3.5 cm AAA at baseline, based on intervening at a fixed 5.5 cm threshold versus no intervention at all

Age	Expected life‐years remaining		Difference
	Given intervention at 5.5 cm	Given no intervention	
65	15.526	13.046	2.480
70	12.113	10.774	1.339
75	9.182	8.561	0.621
80	6.801	6.561	0.240
